# Oil recovery for fractured-vuggy carbonate reservoirs by cyclic water huff and puff: performance analysis and prediction

**DOI:** 10.1038/s41598-019-51807-4

**Published:** 2019-10-23

**Authors:** Daigang Wang, Jingjing Sun

**Affiliations:** 1 Beijing International Center for Gas Hydrate, Peking University, Beijing, 100871 China; 2 Key Laboratory of Unconventional Oil & Gas Development, China University of Petroleum (East China), Ministry of Education, Qingdao, 266580 China; 3 Research Institute of Petroleum Exploration and Development, PetroChina, Beijing, 100083 China

**Keywords:** Crude oil, Mechanical engineering

## Abstract

Cyclic water huff and puff (CWHP) has proven to be an attractive alternative to improve oil production performance after depletion-drive recovery in fractured-vuggy carbonate reservoirs. However, due to the impact of strong heterogeneity, multiple types of fractured-vuggy medium, poor connectivity, complex flow behaviors and oil-water relationship, CWHP is merely suitable for specific types of natural fractured-vuggy medium, usually causing a great difference in actual oil-yielding effect. It remains a great challenge for accurate evaluation of CWHP adaptability and quantitative prediction of production performance in fractured-vuggy carbonate reservoir, which severely restricts the application of CWHP. For this study, we firstly enable the newly developed fuzzy grey relational analysis to quantify the adaptability of CWHP. With production history of several targeted producers, the accuracy of the proposed method is validated. Based on the traditional percolation theory and waterflood mechanisms in various types of fractured-vuggy medium, a quantitative prediction model for cyclic water cut *f*_wp_ and increased recovery factor Δ*R* is presented. The CWHP production performance is discussed by using the Levenberg-Marquardt algorithm for history matching. With a better understanding of the *f*_wp_ ~ Δ*R* curve characteristics in different types of fractured-vuggy medium, proper strategies or measures for potential-tapping remaining oil are provided. This methodology can also offer a good basis for engineers and geologists to develop other similar reservoirs with high efficiency.

## Introduction

Global hydrocarbon resources stored in different types of carbonate rocks are particularly abundant, and the proven recoverable reserves are up to 1.8 trillion barrels, which play a very significant role in energy supply^1^. To our best of knowledge, The carbonate reservoirs can be mainly divided into three categories: porous type, fractured-porous type and fractured- vuggy type^2–7^. In recent years, China has made several great breakthroughs to effective development of fractured-vuggy Carbonate reservoirs, e.g., Tahe and Tarim oilfields^8^. Due to great original oil in place (OOIP), the fractured-vuggy carbonate reservoirs distributed in Tarim basin have drawn much attention from oil industry and scholars.

**Figure 1:**
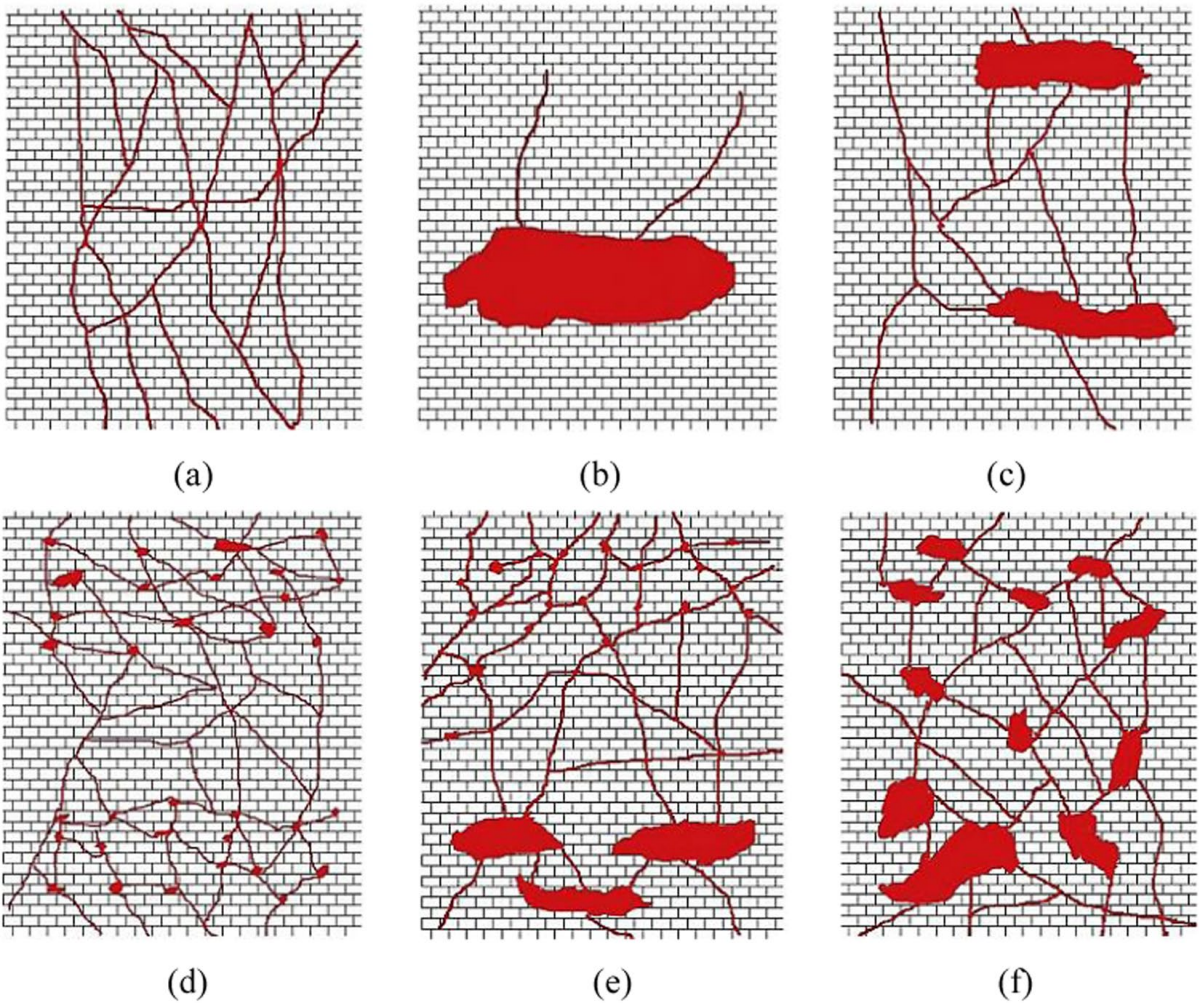
Different patterns of fractured-vuggy medium (Lyu *et al*.^13^). (**a**) fracture network; (**b**) isolated cave; (**c**) cave-fracture-cave; (**d**) pore-fracture-pore; (**e**) pore-fracture-cave; (**f**) cave network.

Compared with porous and fractured-porous carbonate reservoirs, the fractured-vuggy carbonate reservoirs are usually subjected to strong heterogeneity, multiple types of reservoir bodies, poor connectivity, complicated flow dynamics and various oil-water relationship. Based on the investigation of Wang *et al*.^9^, the effective development for fractured-vuggy carbonate reservoirs should be based on identification and characterization of individual fractured-vuggy units. There are a great variety of reservoir space types and compound patterns between each other, as shown in Fig. 1. Large isolated caves, eroded fracture-vugs and dissolved fractures are the main storage spaces and flow channels, indicating complicated multi-scale distribution characteristics. Previous studies showed that the fractured-vuggy carbonate reservoirs are largely dependent on primary depletion and natural water flooding for exploration, and the oil recovery factor is quite low with only 6.0%~8.0%^10^.

Note that, all the above-mentioned conditions can lead to a heterogeneous spatial distribution of remaining oil in fractured-vuggy carbonate reservoirs. In order to improve oil production performance as much as possible, Wang *et al*.^11,12^ and Yuan *et al*.^13^ carried out laboratory experiments to analyze the residual oil characteristics in fractured-vuggy carbonate reservoirs after water flooding and N_2_ flooding by creating specific physical models. Starting from the practice situation of Tahe oilfield, and integrated dynamic evaluation of depletion drive performance, Rong *et al*.^14^ put forward seven major patterns and thirteen sub-classes of remaining oil distribution. The potential-tapping strategies were further presented for various remaining oil distribution patterns. In addition, to tackle the drawbacks of rapid production decline and low recovery rate caused by weak natural energy and sharp water cut rise, many researchers also made great attempts to investigate the feasibility of cyclic water huff and puff (CWHP)^15,16^, N_2_ stimulation^17^, N_2_ flooding^12,18,19^ and N_2_-based foam^20^ to enhance oil recovery for fractured-vuggy carbonate reservoirs, as they believed that there should be a certain EOR method suitable for this type of reservoir. The CWHP was proven to be one of the most efficient techniques to improve oil production performance during the late stage of depletion-drive recovery mainly because of its high input-output ratio and relatively strong flexibility. However, pilot tests indicated that, the CWHP was not adapted to all types of fractured-vuggy bodies^10^. With the increase of water injection during CWHP, the oil- yielding effect tends to be worse, and more and more CWHP producers are ineffective, thus causing large amount of remaining oil unexploited at upper positions of producers. Moreover, the coexistence of porous and free-flow domains over a wide range of scales may result in inaccurate description of the complicated fluid flow behaviors in fractured-vuggy media with experimental and theoretical studies in most cases^21–23^. How to resolve the adaptability evaluation of the CWHP and understand the actual production performance in various types of fractured-vuggy bodies in order to search suitable potential-tapping strategies or measures remain a great challenge to all of us^24,25^.

In last decades, numerous statistical approaches were proposed to investigate various physical, chemical and biological processes, in which the GRA (Grey Relational Analysis) is treated as an attractive tool to measure the degree of approximation among the data sequences with a dimensionless grey relational grade. The GRA^26^ denotes a grey system theory that evaluates the complicated intrinsic relationship between one influential parameter and the others in a specified system. The absolute difference between data sequences is essentially measured to investigate the approximate relationship among sequences with less data. The GRA technique is also employed to understand the synergistic effects of various parameters in order to overcome the shortcomings encountered by other statistical methods^27^. The GRA has a great advantage to determine the intrinsic relationships within less data sequences controlled by various influential factors. Although the GRA technique was successfully used for various areas, e.g., engineering practice^28–30^, medicine^31^, hydrocarbon recovery^32–34^ and microbial production^35–37^. To our best knowledge, few studies used this method to study the adaptability of CWHP in fractured-vuggy carbonate reservoirs.

The goal of this study is to understand actual oil recovery in naturally fractured-vuggy carbonate reservoir by combining grey relation analysis of CWHP adaptability and production performance prediction of CWHP in various types of fractured-vuggy units, which offers a deep understanding for remaining oil potential after depletion-drive recovery and a reliable basis for efficient EOR strategies in similar reservoirs.

## Grey Relational Analysis for CWHP Adaptability

Due to lack of accurate evaluation on the potential of remaining oil after depletion-drive recovery in fractured-vuggy carbonate reservoirs, it is of great difficulty to screen the best potential producers for CWHP. In this work, taking the synergistic effects of drilling data, geological features, dynamic evaluation results and depletion-drive production responses into account, an integrated evaluation index system for CWHP adaptability in fractured-vuggy carbonate reservoirs is firstly developed to improve the decision-making ability of potential tapping. Thereafter, fuzzy grey relational analysis is performed to deal with the problem with high uncertainty.

### Evaluation index system of CWHP adaptability

To achieve an accurate evaluation of CWHP adaptability in fractured-vuggy carbonate reservoirs, it is important to consider the synergistic impacts of various static and dynamic data obtained from pilot tests, mainly including the drilling data, geological features, dynamic evaluation results and depletion-drive production responses. A static-dynamic evaluation index system for CWHP adaptability is firstly developed. Table 1 also shows the effect of various evaluation indexes on adaptability of the CWHP. In regard to a targeted fractured- vuggy reservoir with large leakage liquid volume, it is more possible to achieve a higher oil production by cyclic water huff and puff. As the aquifer volume increases, the bottom-water coning during CWHP is usually faster, thus resulting in a more rapid water breakthrough and shorter duration time of primary recovery.

**Table 1:** Static-dynamic evaluation index system and criterion for CWHP adaptability.

Investigated factor		Evaluation criterion for CWHP adaptability		
		Suitable	Relatively suitable	Unsuitable
Drilling data	leakage liquid volume (m3)	Large leakage	A little	No leakage
	Drilled position	Upper	middle	lower
Geological features	Types of reservoir body	Cave network	Fracture-vug	Dissolved fissures
	Space distribution	Vertically distributed	Uniformly distributed	Laterally distributed
	Sealing degree	good	poor	bad
Reservoir dynamic evaluation results	Well-recoverable reserves (10^4^t)	15∼25	5∼15	>25 or <5
	Drive energy	Elastic drive	Weak waterflood	Strong waterflood
	Aquifer volume (10^4^m3)	little	poor	strong
Depletion-drive Production response	Law of water breakthrough	No water influx	Wave-like	Stair-rising or abrupt coning
	Recovery rate(%)	>10	5 ∼ 10	<5
	Water cut (%)	<20	20 ∼ 60	>60

**Figure 2:**
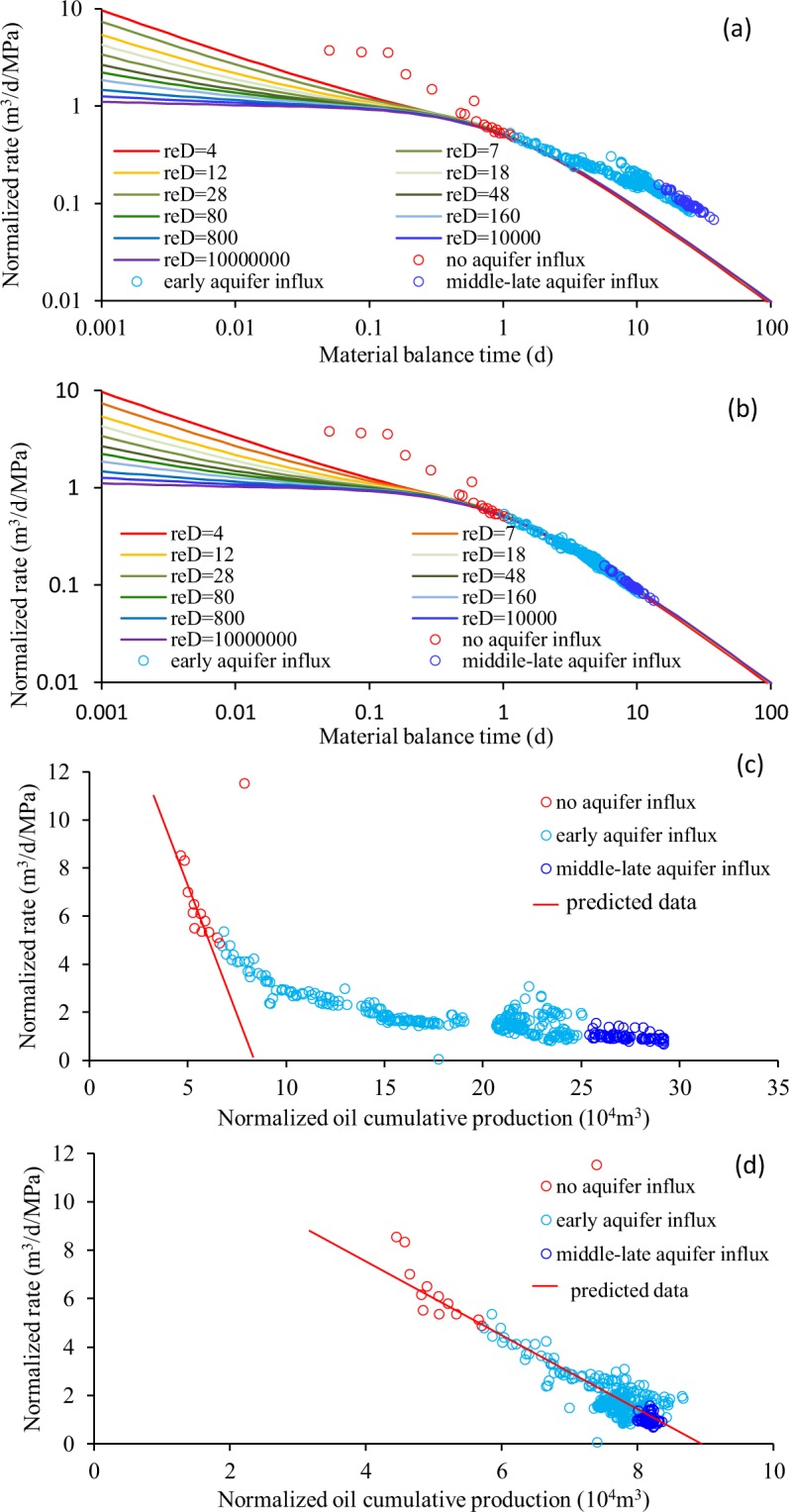
The curve-fitting process for estimating well-recoverable reserve and aquifer volume in a targeted CWHP producer, respectively: (**a**,**b**) Blasingame type curve; (**c**,**d**) FMB type curve.

Figure 2 shows the curve-fitting procedures for estimating the well-recoverable reserve and aquifer volume to a targeted producer in fractured-vuggy reservoir. Taking the impact of production rate and well bottom-hole pressure into account, we select the Blasingame^38^ type curve and flowing material balance (FMB) method^39^ for preliminary evaluation. Thereafter, an analytical radial model is developed for single-well production history matching in order to obtain a favorable estimation of well-recoverable reserve and aquifer volume.

To quantify the CWHP adaptability in fractured-vuggy carbonate reservoirs, it is critical to evaluate the impact of various controlling factors and normalize the original data sequences to alleviate the dimension inconsistency. Table 2 displays the ranges of various controlling factors and the normalization of targeted producer’s original data sequences. The symbol ‘↗’ denotes to a positive effect of investigated factors on CWHP adaptability, whereas the symbol ‘↘’ shows a negative impact on the CWHP adaptability.

**Table 2:** Value ranges of influential factors and normalization of original data sequences.

Num.	Symbol	Influential factor	Value range	Trend	Targeted producer	Normalization
1	C1	leakage liquid volume (m3)	[0, 100]	↗	80.0	0.8
2	C2	Drilled position	[0, 1]	↗	0.9	0.9
3	C3	Reservoir body type	[0, 100]	↗	90.0	0.9
4	C4	Space distribution	[0, 1]	↗	0.8	0.8
5	C5	Sealing degree	[0, 100]	↗	95.0	0.95
6	C6	Well-recoverable reserves (10^4^t)	[0, 25]	↗	5.0	0.2
7	C7	Drive energy	[0, 100]	↘	60.0	0.4
8	C8	Aquifer volume (10^4^m3)	[0, 100]	↘	40.0	0.6
9	C9	Law of water breakthrough	[0, 100]	↘	10.0	0.9
10	C10	Recovery rate before water injection (%)	[0, 10]	↗	8.0	0.8
11	C11	Water cut after depletion (%)	[0, 100]	↘	40.0	0.6

### Fuzzy grey relational analysis

Grey relational analysis (GRA) is an efficient statistical technique for determining the degree of approximation among data sequences with a dimensionless grey relational grade. However, Yazdani *et al*.^40^ confirmed that, the usually considered 0.5 of resolving coefficient in the traditional GRA is often inadequate to address the ambiguity found in the available information, as well as the critical fuzziness in decision judgement and preference. It is more suitable to characterize the degree of data certainty using an interval. Therefore, to evaluate the adaptability of CWHP in fractured-vuggy carbonate reservoirs, the newly developed fuzzy grey relational analysis (FGRA)^41^ is adopted. The procedures are illustrated as follows.

Step 1: Establish the reference matrix and comparison matrix. The reference matrix is described as

1 $${{\boldsymbol{Y}}}_{0}=[{Y}_{0}(1),{Y}_{0}(2),\ldots ,{Y}_{0}({\rm{n}})]$$

where the reference matrix denotes a presumed criterion or optimal values of the investigated factors. If the production performance is better as each factor increases, the maximum value of data sequence is regarded as the reference criterion, otherwise, the reference variable is equal to the minimum value of data sequence. If the dimension of original sequences is *m* and the CWHP adaptability is investigated under *n* different influential factors, so the comparison matrix is given by:

2 $${\boldsymbol{Y}}=[\begin{array}{c}{{\boldsymbol{Y}}}_{0}\\ {{\boldsymbol{Y}}}_{1}\\ \begin{array}{c}\vdots \\ {{\boldsymbol{Y}}}_{m}\end{array}\end{array}]=[\begin{array}{ccc}\begin{array}{c}{Y}_{0}(1)\\ {Y}_{1}(1)\\ \begin{array}{c}\vdots \\ {Y}_{m}(1)\end{array}\end{array} & \begin{array}{c}{Y}_{0}(2)\\ {Y}_{1}(2)\\ \begin{array}{c}\vdots \\ {Y}_{m}(2)\end{array}\end{array} & \begin{array}{cc}\begin{array}{c}\ldots \\ \ldots \\ \begin{array}{c}\vdots \\ \ldots \end{array}\end{array} & \begin{array}{c}{Y}_{0}(n)\\ {Y}_{1}(n)\\ \begin{array}{c}\vdots \\ {Y}_{m}(n)\end{array}\end{array}\end{array}\end{array}]$$

Step 2: Normalize the original data sequences. As the influential factors and the reference values have great dimensions, the following equation should be used for normalization

3 $${Y}_{i}(k)=\frac{{Y}_{i}(k)-\,{\rm{\min }}\,{Y}_{i}(k)}{{\rm{\max }}\,{Y}_{i}(k)-\,{\rm{\min }}\,{Y}_{i}(k)},\,i=0,1,\ldots ,m;k=1,2,\ldots ,n$$

Step 3: Estimate the cosine value of fuzzy membership. For this study, the included- angle cosine method is used, which is not influenced by the linearly proportional data relationship. The similarity of the two factors is achieved according to the value of the included-angle cosine. It is computed as follows:

4 $${r}_{1}=\frac{{\sum }_{k=1}^{n}{Y}_{0}(k)Y(k)}{\sqrt{{\sum }_{k=1}^{n}{Y}_{0}{(k)}^{2}}\sqrt{{\sum }_{k=1}^{n}Y{(k)}^{2}}}$$

Step 4: Determine the grey relational coefficient *ξ*_ik_.

5 $${\xi }_{{\rm{ik}}}=\frac{{\Delta }_{{\rm{\min }}}+{{\rm{\psi }}\Delta }_{{\rm{\max }}}}{\Delta (k)+{{\rm{\psi }}\Delta }_{{\rm{\max }}}}$$

6 $${\Delta }_{{\rm{\min }}}={\min }_{i}\,{\min }_{k}|{Y}_{0}(k)-{Y}_{i}(k)|$$

7 $${\Delta }_{{\rm{\max }}}={\max }_{i}\,{\max }_{k}|{Y}_{0}(k)-{Y}_{i}(k)|$$

where ψ denotes the resolving coefficient, [0, 1]. The resolving coefficient must satisfy the integrity and anti-interference of the relational coefficient due to that a large or small value is unable to describe the intrinsic relationship of influential factors. The following procedure is used to obtain the favorable resolving coefficient:

(a) Compute the mean value of all absolute difference $\overline{\Delta }$:

8 $$\overline{\Delta }=\frac{1}{m\cdot n}{\sum }_{i=1}^{m}{\sum }_{k=1}^{n}|{Y}_{0}(k)-{Y}_{i}(k)|$$

(b) Based on the ratio $c=\overline{\Delta }/{\Delta }_{{\rm{\max }}}$, the resolving coefficient is described as.

9 $${\rm{\psi }}\in \{\begin{array}{ll}[c,1.5c], & c < 1/3\\ 1.5c,2c & c\ge 1/3\end{array}$$

For this study, if c < 1/3, *ψ* = 1.25c, else if c ≥ 1/3, *ψ* = 1.75c.

Step 5: Obtain the Euclidean distance between the reference and comparison matrice, and define the weight of influential factors in the reference matrix using the analytical hierarchy process.

10 $${\boldsymbol{W}}=[{w}_{1},{w}_{2},\,\ldots \,,{w}_{n}]$$

Then, the Euclidean grey relational grade *r*_2_ is obtained by the following formula:

11 $${r}_{2}=1-2\sqrt{{\sum }_{k=1}^{n}{[{w}_{k}(1-{\xi }_{{\rm{ik}}})]}^{2}}$$

Step 6: Determine fuzzy grey relational grade using the fuzzy membership coefficient and the Euclidean grey relational grade, which can be described as

12 $$R=\sqrt{\frac{{{r}_{1}}^{2}+{{r}_{2}}^{2}}{2}}$$

Step 7: Using the magnitude of fuzzy grey relational grades, the adaptability of CWHP in different single-well fractured-vuggy units is finally evaluated.

Table 3 lists the estimated weight values of influential factors during the evaluation of CWHP adaptability according to analytical hierarchy process (AHP). It indicates that, the dynamic production behaviors during depletion-drive recovery usually have a remarkable impact on the adaptability of CWHP. With respect to a typical fractured-vuggy unit having low water cut before water injection, large well-recoverable reserves and small aquifer volume, it is highly efficient to perform CWHP for further potential tapping.

**Table 3:** The estimated weight values of various influential factors based on AHP.

1^st^-layer factor	B1		B2			B3			B4		
**weight**	**0.0374**		**0.1818**			**0.2003**			**0.5805**		
**2** ^**nd**^ **-layer factor**	**C1**	**C2**	**C3**	**C4**	**C5**	**C6**	**C7**	**C8**	**C9**	**C10**	**C11**
weight	0.83	0.17	0.48	0.11	0.41	0.07	0.47	0.47	0.45	0.091	0.45
Total weight	0.06	0.01	0.07	0.017	0.06	0.03	0.18	0.18	0.18	0.04	0.18

On the basis of the magnitude of the fuzzy grey relational grades, the adaptability of CWHP in typical single-well fractured-vuggy units are further ranked into four different categories, i.e., excellent, good, poor and bad, denoting to ranges [0.75, 1.0], [0.5, 0.75], [0.25, 0.5] and [0, 0.25], respectively.

**Figure 3:**
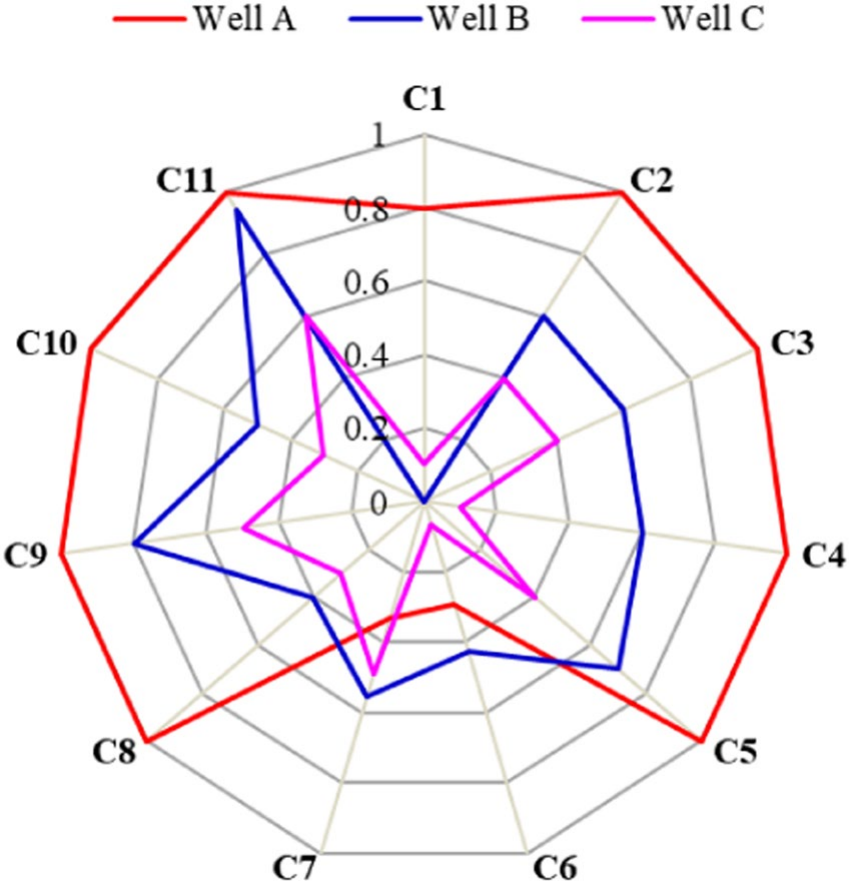
Fuzzy grey relational analysis for CWHP adaptability of three targeted producers.

To validate the accuracy of FGRA, the dynamic and static data of three CWHP producers in a fractured-vuggy carbonate reservoir are used to evaluate the adaptability of CWHP. Table 4 lists the values of influential factors and the estimated fuzzy grey relational grades with respect to three targeted producers. The evaluation results are also shown in Fig. 3.

**Table 4:** Value of influential factors to targeted producers and relevant evaluation results.

symbol	Influential factor	Value of influential factors to targeted producers					
		A		B		C	
C1	leakage liquid volume (m^3^)	large	80	very little	0	minor	10
C2	Drilled position	upper	1.0	middle	0.6	middle	0.4
C3	Types of reservoir body	cave network	100	fracture-vug	60	fracture-vug	40
C4	Space distribution	well- distributed Vertically	1.0	poor- distributed vertically	0.6	well- distributed laterally	0.1
C5	Sealing degree	good	100	poor	70	bad	40
C6	Well-recoverable reserves (10^4^t)	7.3	7.3	10.7	10.7	1.7	1.7
C7	Drive energy	elastic drive	0	weak waterflood	40	weak waterflood	70
C8	Aquifer volume (10^4^m3)	very little	0	very little	20	poor	50
C9	Law of water breakthrough	no water influx	0	wave-like	40	stair-rising	70
C10	Recovery rate before water injection (%)	3.32	3.32	5.56	5.56	5.11	5.11
C11	Water cut after depletion (%)	0	0	5.5	5.5	26.62	26.62
Grey relational grade		0.784		0.552		0.474	
Evaluation result		excellent		good		poor

It can be seen from Fig. 3, the larger the area restricted by broken lines, the higher the adaptability of CWHP in single-well fractured-vuggy units. In practice, the cumulative oil production of typical CWHP producers A, B and C are 3.51 × 10^4^t, 3754t and 1433.2t, respectively, which is consistent with the result of fuzzy grey relational analysis, indicating that the static-dynamic evaluation index system and the FGRA method are efficient enough to help decision-making of the best CWHP wells for further potential-tapping of remaining oil in fractured-vuggy carbonate reservoir.

## Performance Prediction for CWHP

Due to the impact of strong heterogeneity, various types of fractured-vuggy bodies, poor connectivity, multiscale flow behaviors and complicated water-oil relationship, CWHP is merely suitable for specific types of fractured-vuggy bodies, thus causing a great difference in actual production performance. Moreover, with the increasing of water injection during the CWHP, it may suffer from problems of rapid production decline and abrupt increase of water cut, and large amount of remaining oil are still around the top of producers. Therefore, it is essential to quantify the uncertainty and complexity of CWHP production performance in order to explore the proper potential-tapping strategies. For this study, based on the traditional percolation theory and waterflood mechanisms for fractured-vuggy bodies, a quantitative prediction model of cyclic water cut and increased recovery factor is proposed. By using the L-M algorithm for history matching, the actual CWHP production performance for different types of fractured-vuggy medium are further investigated.

### Quantitative prediction model for CWHP

With regard to traditional waterflooding reservoirs, the widely used method to describe the relation for oil/water relative permeability ratio and water saturation is the Craft equation, which can be stated as

13 $$\mathrm{ln}\,\frac{{K}_{ro}}{{K}_{rw}}=a+b{S}_{w}$$

where *K*_ro_ and *K*_rw_ are the oil-phase and water-phase relative permeability, respectively; *S*_w_ is the water saturation; *a* and *b* are the unknown parameters.

However, previous studies indicate that, with respect to ultra-high water cut period, there exists a great deviation between the predicted performance by the traditional Craft empirical model and the observed data^42^, which severely restricts the application of this method. To overcome the inherent drawback, an improved Craft relationship was developed as follows

14 $$\mathrm{ln}\,\frac{{K}_{ro}}{{K}_{rw}}=a+b{S}_{w}+c{{S}_{w}}^{2}+d{{S}_{w}}^{3}$$

The generalized Darcy’s law and water fractional flow equation are described as.

15 $$v=\frac{K}{\mu }\frac{dp}{dz}{[1-\frac{1}{1+a{(dp/dz)}^{b}}]}^{4}$$

16 $${f}_{w}=\frac{{Q}_{w}}{{Q}_{w}+{Q}_{o}}=\frac{{K}_{rw}{\mu }_{o}}{{K}_{rw}{\mu }_{o}+{K}_{ro}{\mu }_{w}}$$

As for single-well fractured-vuggy units, the increased recovery factor by CWHP can be computed as

17 $$\Delta R=\frac{{V}_{p}({S}_{w}-{S}_{w0}){\rho }_{o}}{N{B}_{o}}$$

where Δ*R* is the increased recovery factor; *V*_p_ is the pore volume, m^3^; *ρ*_o_ is the density of crude oil, kg/m^3^; *N* is the original oil in place (OOIP), t; *B*_o_ is the volume coefficient of crude oil; *S*_w0_ is the water saturation of fractured-vuggy body during the late stage of primary depletion; *S*_w_ is the water saturation after CWHP.

With regard to a specific fractured-vuggy reservoir, the pore volume, the density of crude oil, the original oil in place (OOIP) and water saturation after depletion-drive recovery are usually constant, and the increased recovery factor is positively proportional to the water saturation after CWHP, i.e., Δ*R* ∞ *S*_w_. Therefore, the quantitative prediction model of cyclic water cut *f*_wp_ and increased recovery factor Δ*R* is described in the following.

18 $${f}_{wp}=\frac{1}{1+{e}^{a+b\Delta R+c\Delta {R}^{2}+d\Delta {R}^{3}}}$$

where *a*, *b*, *c* and *d* are the unknown model parameters, respectively.

To obtain a satisfactory history matching of the observed production data, a least-square objective function is established, and takes the form of Eq. (19).

19 $$O({\bf{m}})=\frac{1}{2}{({\boldsymbol{g}}({\boldsymbol{m}})-{{\boldsymbol{d}}}_{{\rm{obs}}})}^{T}{{{\boldsymbol{C}}}_{D}}^{-1}({\boldsymbol{g}}({\boldsymbol{m}})-{{\boldsymbol{d}}}_{{\rm{obs}}})$$

where *O*(**m**) denotes the objective function; **m** denotes a (n × 1) vector of the controlling parameters; *T* is a symbol indicating the transpose of vector or matrix; **d**_obs_ denotes a (n × 1) vector of observed data; ${\boldsymbol{g}}({\boldsymbol{m}})$ denotes a (n × 1) vector of predicted data; ${{\boldsymbol{C}}}_{D}$ denotes the (n × n) covariance matrix of data.

In this paper, optimization is performed using the Levenberg-Marquardt (L-M) algorithm. Besides, a finite difference method is employed to calculate the gradient of objective function at unknown controlling parameters. When using the L-M algorithm to investigate a least-square history matching problems, smooth transmission is usually achieved between the steepest descent algorithm and Newton algorithm. If the least-square objective function is far from the optimal solution, the convergence direction is close to that obtained by the steepest descent algorithm. Otherwise, the convergence direction will be identical to that of the Newton algorithm.

The iteration computation scheme to solve the least-square problem using the L-M algorithm is stated as follows.

20 $$(\lambda {\boldsymbol{I}}+{\bf{H}}({{\bf{m}}}^{k})){\rm{\delta }}{{\bf{m}}}^{k+1}=-\,\nabla O({{\bf{m}}}^{k})$$

where *λ* is the damping factor; ${\bf{H}}({{\bf{m}}}^{k})$ is the symmetric, half-positive Hessian matrix; ***I*** is an identity matrix; δ**m**^k+1^ is the difference matrix between *k*th and (*k* + 1)th iteration.

**Figure 4:**
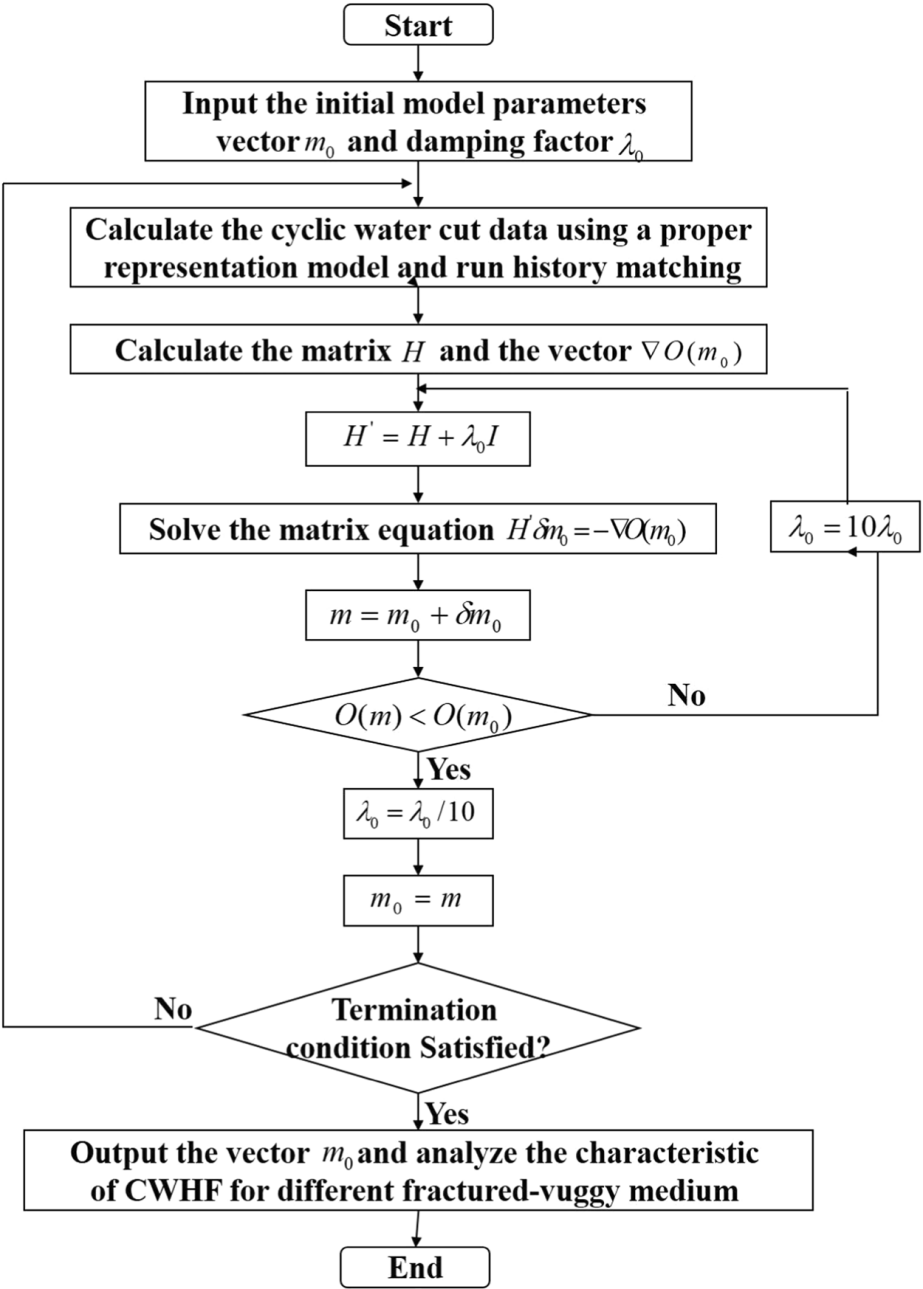
Calculation procedure of analyzing CWHP production performance based on the proposed quantitative prediction model in fractured-vuggy carbonate reservoirs.

The following describes how the L-M algorithm is used for optimization of a least- square objection function. First, input the initial damping factor *λ*_0_. When each iteration is over, it is key to update the damping factor. The basic principle is presented as follows: (1) Calculate the vector of unknown controlling parameters **m**^k+1^. If *O*(**m**^k+1^) ≥ *O*(**m**^k^), the iteration is treated as a failure, and then *λ* = *λ* × 10. If *O*(**m**^k+1^) < *O*(**m**^k^), the iteration is successful, and then *λ* = *λ*/10. (2) Input the updated damping factor *λ* into Eq. (20) and perform the next iteration. The above-mentioned iteration is repeated until the termination condition is ultimately satisfied. Figure 4 displays the detailed calculation procedure of analyzing the actual CWHP production performance in fractured-vuggy carbonate reservoirs based on the proposed *f*_wp_ ~ Δ*R* quantitative prediction model.

## Results and Discussion

**Figure 5:**
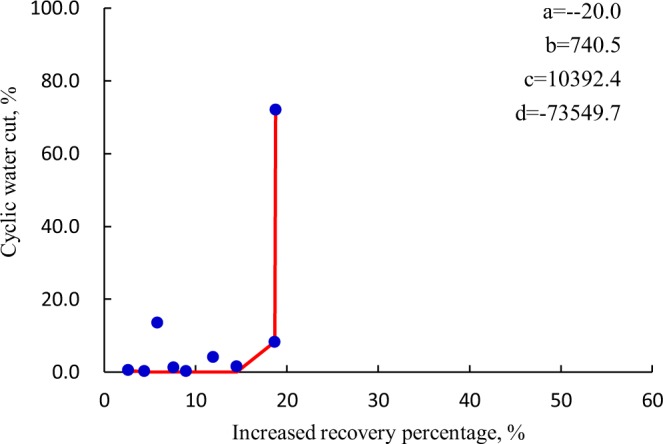
The concave profile of *f*_wp_ ~ Δ*R* curve with respect to karst caves.

Based on production history of CWHP in typical fractured-vuggy carbonate reservoir founded in Tarim basin, the proposed prediction model for *f*_wp_ ~ Δ*R* is used to understand the characteristics of cyclic water huff and puff in various types of fractured-vuggy reservoir bodies. Figure 5 shows the ultimate curve fitting effect of typical karst cave. It demonstrates that, the *f*_wp_ ~ Δ*R* curve of karst caves seems like a concave profile, denoting to a satisfactory outperformance. Proper strategies are preferable to enlarge the water-free production period and inhibit the sharp bottom-water conning as long as possible. Once water breakthrough is achieved, oil production becomes worse. When the CWHP is ineffective, the nitrogen-based huff-n-puff has proven to be an attractive alternative to displace the attic remaining oil around the top of karst caves.

**Figure 6:**
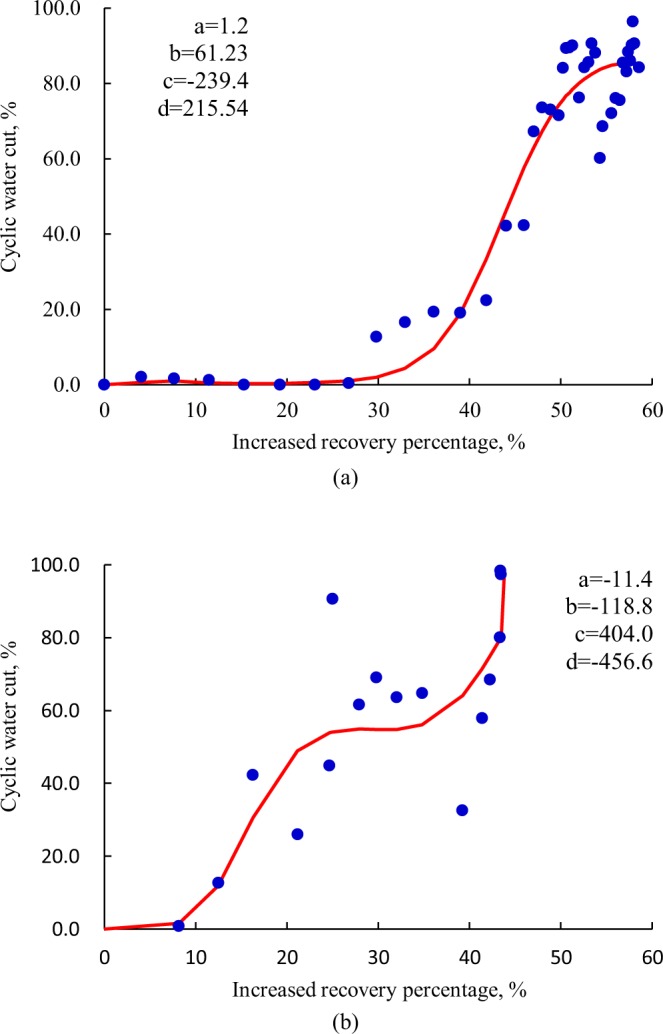
The *f*_wp_ ~ Δ*R* curve of eroded pores and karst curves. (**a**) *S*-shaped type; (**b**) Stair-rising type.

With respect to eroded pores and caves, the *f*_wp_ ~ Δ*R* curve during cyclic water huff and puff obeys a S-shaped or stair-rising law, as shown in Fig. 6, which indicates a relatively great oil production and long water-free production period. Majority of remaining oil unexploited around the top of producers will be recovered after water breakthrough due to the strong bottom water support. During the later stage of production, shut-in to inhibit water coning or water drainage-oil production technique is preferable to improve the CWHP development effect with the greatest effort.

**Figure 7:**
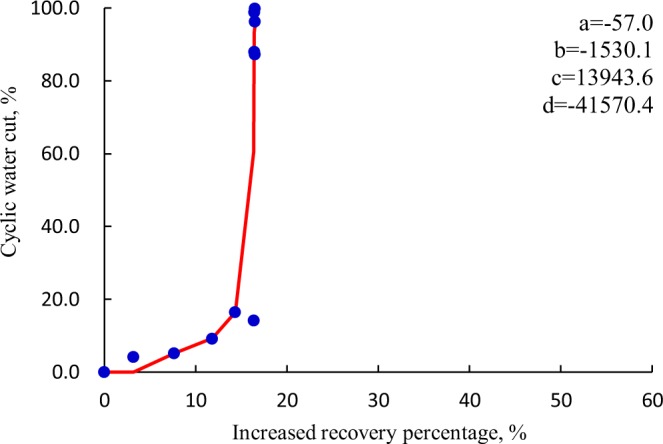
The down-convex profile of *f*_wp_ ~ Δ*R* curve with respect to dissolved pores and fissures.

As for the dissolved pores and fissures, the *f*_wp_ ~ Δ*R* curve usually conforms to a down-convex law, as shown in Fig. 7. Rapid production decline and low recovery rate are obtained due to weak natural energy and rapid water cut rise. Once water breakthrough is achieved, oil production rate will decrease sharply. The producers for CWHP are under long-time interval production, and the effect of shut-in to control the speed of water conning is negligible in most cases. The unconventional potential-tapping measures, such as large- scale acid fracturing-hydraulic dilation, sidetracking, and nitrogen-based huff-n-puff are recommended for this type of fractured-vuggy medium.

## Conclusions

In order to tackle the drawback of inaccurate evaluation on potential of the remaining oil after depletion-drive recovery in fractured-vuggy carbonate reservoirs, we enable the newly developed fuzzy grey relational analysis to quantify the adaptability of CWHP in different types of single-well fractured-vuggy units. Using the production history of several targeted producers, the accuracy of the proposed method is validated, which is of great importance to improve the decision-making ability of potential-tapping.

Based on the traditional percolation theory and waterflood mechanisms in different types of fractured-vuggy medium, a quantitative prediction model between cyclic water cut *f*_wp_ and increased recovery factor Δ*R* is presented. The actual production performance of CWHP producers are further analyzed. Results show that, the *f*_wp_ ~ Δ*R* curve of karst caves seems like a concave profile, indicating a satisfactory outperformance. Proper strategies are preferable to enlarge the water-free production period and inhibit bottom-water conning. When water breakthrough is achieved, oil production rate will decline sharply. As for eroded pores and karst caves, the *f*_wp_ ~ Δ*R* curve usually obeys a S-shaped or stair-rising law. Large amount of remaining oil unexploited around the top of producers are recovered after breakthrough due to the strong bottom water support. With respect to the dissolved pores and fissures, it usually satisfies a down-convex law. The producers are under long-time interval production, and the effect of shut-in to inhibit water conning is negligible in most cases.

## Data Availability

All raw data can be uploaded to the submission system if needed.
